# Access to paediatric emergency departments in Italy: a comparison between immigrant and Italian patients

**DOI:** 10.1186/1824-7288-35-3

**Published:** 2009-02-22

**Authors:** Erica Clara Grassino, Carla Guidi, Alice Monzani, Pasquale Di Pietro, Gianni Bona

**Affiliations:** 1 Department of Pediatrics, AOU Maggiore della Carit, University of Piemonte Orientale, Novara, Italy; 2 Emergency Room and Emergency Medicine Division G. Gaslini Institute, Genova, Italy

## Abstract

**Objective:**

The aim of the study was to investigate whether access to paediatric emergency departments differed between foreign and Italian patients.

**Methods:**

We performed a cross-sectional study between January-December 2007 to analyse attendance's characteristics in the paediatric emergency departments of ten Italian public hospitals. The study population included each foreign patient and the following Italian patient admitted to the same emergency department. All causes of admission of these subjects were evaluated, together with the child's age, gender, country of birth, parents' nationality, time of admission, severity code and discharge-related circumstances.

**Results:**

We enrolled 4874 patients, 2437 foreign (M:F = 1409:1028) and 2437 Italian ones (M:F = 1368:1069). Most of foreign and Italian patients' admissions were sorted as green (72.5% and 87.8%, respectively) or white codes (25.2% and 9.8%, respectively). The most frequent causes for attendance concerned respiratory tract diseases, followed by gastroenteric ones and injuries in both groups.

**Conclusion:**

In our survey immigrants didn't access to emergency departments more than Italian children. Both of them referred to emergency departments mainly for semi-urgent or non-urgent problems. Foreign and Italian patients suffered from the same pathologies. Infectious diseases traditionally thought to be a potential problem in immigrant populations actually seem to be quite infrequent.

## Background

Italy has traditionally been a country of emigration, with immigration being a relatively recent phenomenon beginning in the mid-eighties. Nowadays, immigrants in Italy are 3.432.651, representing 5.8% of total population [1]. Mainly, immigrants come from developing countries that are economically disadvantaged compared with Italy. One of the most worrisome political issues regarding immigration is the ability of healthcare institutions to serve the increasing number of immigrants and to provide equity of access to healthcare services among this population. The Italian National Health System provides universal access to healthcare; nevertheless, often the utilisation of primary and specialized care might present several barriers for foreign people, including lack of knowledge about how to access these services and difficulties in making an appointment (language, time schedules, waiting lists, etc.). Therefore, it is possible that foreign people tend to access to emergency departments more easily than to primary and specialised care services. It has to be taken into account that economic indicators of poverty have been related to poorer health and higher emergency units utilisation [2]. Previous studies describing healthcare services use by immigrants tended to highlight their overall under-utilisation of services compared with non-immigrants, the preponderant access to public units over the private sector, and the greater use of emergency rooms to counteract access barriers to other health services [3-5]. Few studies analysing worldwide paediatric healthcare services utilisation showed a similar disadvantaged condition for foreign children, too, resulting in an excessive and often improper use of emergency units [6,7]. Up to now, paediatric emergency services utilisation in Italy has not yet been studied in depth. We aimed to quantify the amount of accesses to emergency units by immigrant and Italian population analysing one-year activity of ten Italian public paediatric hospitals. Therefore, we evaluated admissions to paediatric emergency units of foreign and Italian children, in order to test the hypothesis that the emergency department utilisation would be higher, and consequently often inappropriate, among immigrants than among the host population. Secondary aim was to identify the causes of consultation for foreign and Italian patients and to verify whether immigrants suffer from specific import diseases.

## Methods

Between January and December 2007 a multicentric cross-sectional study was undertaken in the paediatric emergency department of ten Italian public hospitals. Clinical notes for all foreign patients from developing countries outside the European Union admitted to the paediatric emergency departments were recorded by a medical interviewer. A patient was considered as foreign if one or both parents were born outside Italy and the European Union. Each foreign patient was matched with the following Italian patient admitted to the same paediatric emergency department, whose clinical notes were collected, too.

Data about age, gender, country of birth, maternal and paternal nationality, time of admission, cause of consultation and severity code were recorded for each child enrolled. Patients were divided into five groups according to age: newborns if <1 month, infants if 1 month – 1 year, preschool-aged children if 1 – 5 year, school-aged children if 6 – 10 years, adolescents if >11 years. Children and their parents' nationality was classified into nine groups to simplify the analysis: 1. Italy, 2. non-EU Eastern Europe, 3. Latin America, 4. Nile Valley, 5. Northern Africa, 6. sub-Saharan Africa, 7. India, Pakistan and Sri-Lanka, 8. China and the rest of Asia, 9. nomads and Gypsies. Time of admission was recorded as day-time if admission to the emergency department took place between 8 a.m. and 8 p.m. and night-time if between 8 p.m. and 8 a.m. Cause of consultation was synthesized with the main symptom complained at admission. Evaluating the severity of the symptoms complained, patients were sorted into four categories identified by four different colours according to Canadian Pediatric Triage and Acuity Scale Guidelines [8]: white if non-urgent (cases and situations that could also be managed by the family Paediatrician – these patients will be examined only after the most serious cases have been managed), green if semi-urgent (cases with postponable health troubles – there is no risk of death and the patient will be cared for after more urgent cases), yellow if urgent (patients with the risk of fast prejudice of vital functions), red if emergency (cases with immediate risk of death). The diagnosis at discharge was analyzed according to a broad spectrum of variables: respiratory diseases, injuries, gastroenteric diseases, skin affections, fever, localized infections, childhood infectious diseases, genitourinary infections, foreign bodies ingestion or inhalation, poisoning, surgical problems and burns. Discharge-related circumstances (discharge to home, further outpatient investigations, temporary observation, hospital admission, voluntary discharge against the physician's recommendation or death) were also evaluated.

The study has been approved by the local Ethical Committee and informed consent was signed by the parents.

Statistical analysis was performed with SPSS 16.0 software (SPSS Inc, Chicago, IL).

## Results

### Study population

In the period analyzed in the survey, 2437 foreign citizens referred to the paediatric emergency departments of the hospitals taking part to this multicentric study and were enrolled for the survey. As many Italian patients admitted to the same paediatric emergency department were enrolled. Among immigrant patients 1409 (57.8%) were males and 1028 (42.2%) females; among Italian ones, 1368 (56.1%) were males and 1069 (43.9%) females. Out of the 2437 foreign subjects, 35 (1.4%) were newborns, 519 (21.3%) infants, 1110 (45.6%) preschool-aged children, 447 (18.3%) school-aged children and 326 (13.4%) adolescents. Out of the 2437 Italian subjects, 34 (1.4%) were newborns, 336 (13.8%) infants, 1193 (49%) preschool-aged children, 424 (17.4%) school-aged children and 450 (18.4%) adolescents.

Analyzing the country of birth of foreign patients, 51.2% of subjects were born in Italy, 17.4% in non-UE Eastern European countries, 15.3% in Northern Africa, 7% in Latin America, 3% in sub-Saharan Africa, 1.7% in the Nile Valley/Arabian countries, 1.9% in China or other Asian countries, 0.9% in India, Pakistan or Sri-Lanka and 1.6% were nomads or Gypsies. Fathers came in most cases from Northern Africa (30.3%), followed by non-UE Eastern Europe (28%), Latin America (14.4%), sub-Saharan Africa (6.6%), China or other Asian countries (4.2%), the Nile Valley/Arabian countries (3.6%), Indian area (1.9%), Italy (0.7%). 9.4% of fathers had no fixed abode/were nomads; father nationality was unknown in 0.9%. Similarly, among the mothers the most frequent country of origin was Northern Africa (28.1%), followed by non-UE Eastern Europe (27.3%), Latin America (14.5%), sub-Saharan Africa (6.4%), China or other Asian countries (4.1%), the Nile Valley/Arabian countries (3.4%), Indian area (1.8%), Italy (0.7%). 9.1% of mothers had no fixed abode/were nomads; mother nationality was unknown in 4.6%.

### Access characteristics

Both among immigrant and Italian patients most of paediatric emergency department visits took place during day-time, between 8 a.m. and 8 p.m. (76.4% and 78.8%, respectively).

According to the severity of symptoms complained most of foreign patients (72.5%) were assigned a green code (semi-urgent), 25.2% of immigrant patients' admissions were sorted as white codes (non-urgent), 2.1% as yellow codes (urgent) and only 0.2% as red codes (emergency). Among the Italian patients, green codes accounted for 87.8% of total consultations, followed by white codes (9.8%), yellow codes (2.3%) and red codes (0.1%).

Analyzing the diagnosis at discharge, most of foreign patients had respiratory diseases (36.3%); 17.8% showed gastroenteric diseases, 12.8% were injured, 5% had skin affections, 5.9% fever, 4.3% localized infections, 3.2% childhood infectious diseases and 1.5% displayed genitourinary infections. Only a small proportion of foreign patients referred to the emergency department for foreign bodies ingestion or inhalation, poisoning, surgical problems or burns. Similarly, among Italian patients the most frequent diagnosis at discharge was a respiratory disease (32.7%), followed by accidental trauma or injuries (17.4%), gastroenteric diseases (15.1%), skin affections (6.6%), fever (6.2%), localized infections (4%), childhood infectious diseases (2.5%), genitourinary infections (1.9%), and by fewer cases of foreign bodies ingestion or inhalation, poisoning, surgical problems or burns. Given the high rate of respiratory diseases, these were analysed separately considering the subgroup pathologies (Table 1). At the end of consultation process 87% of immigrants were discharged to home, 11.9% were hospitalized. Only 7 (0.3%) foreign subjects required further outpatient investigations and 9 (0.4%) a temporary observation, 11 (0.4%) were voluntary discharged against the physician's recommendation. Among Italian patients, discharge to home occurred in 86.7%, hospital admission in 12.6%, temporary observation in 0.5%, voluntary discharge in 0.1% and further outpatients visits in 0.1%. Both between foreign and Italian patients no death was recorded.

**Table 1:** Respiratory diseases.

RESPIRATORY DISEASES	FOREIGN PATIENTS		ITALIAN PATIENTS	
	n	%	n	%
UPPER-AIRWAYS DISEASES	250	28.2	178	22.3
SINUSITIS	4	0.5	5	0.6
ACUTE OTITIS MEDIA	129	14.6	141	17.7
PHARYNGOTONSILLITIS	252	28.5	200	25.1
LARYNGITIS	36	4.1	44	5.5
INFLUENZAL SYNDROME	39	4.4	16	2.0
BRONCHITIS	64	7.2	94	11.8
BRONCHOSPASM	47	5.3	54	6.8
BRONCHIOLITIS	17	1.9	22	2.8
POLMONITIS	47	5.3	43	5.4
Total	885	100.0	798	100.0

Specific respiratory diagnoses in foreign and Italian patients.

## Discussion

The most relevant result of our survey is the high rate of accesses to paediatric emergency departments for non-urgent or semi-urgent medical problems, both between foreign and Italian patients. Indeed, more than 95% of foreign and Italian children were assigned green or white codes. This finding suggests that a large proportion of the demand for emergency departments utilisation often may be attributed to visits for medical problems that do not require emergency treatment. Accident and emergency departments have been created in hospitals with the primary function of providing immediate care for patients with life-threatening medical conditions, trauma, or injuries, but not to treat minor illnesses or provide primary care. In recent years, the emergency department has increasingly become a major provider of health care and this overcrowding has become problematic. People tend to use emergency department as a substitute for general practitioner to treat minor illness. In the context of limited inpatient hospital resources, it is acknowledged that the phenomenon of non-urgent emergency departments visits, which can be managed alternatively and appropriately in general practice, has raised serious concerns among healthcare planners, both because of its magnitude and because the appropriate utilisation of hospital care can significantly improve health outcomes. In particular, the over-utilisation of emergency departments by paediatric patients has become an important problem that must be solved. Medical, social, economic and psychological factors mainly influence the parents' decision to visit the emergency department rather than manage their children at home, or prior to making an unscheduled visit to an emergency department their demands could have been satisfactorily met by an appropriate visit at a different health care level. Reports in literature indicate similar presenting problems in other studies performed in children attending paediatric accident and emergency departments in USA and UK [9-11], in Malaysia [12] and also in Italy [13-15].

It is noteworthy that in our survey immigrants didn't access to emergency departments more than Italian children, in contrast with evidences reported in previous study [4,16,17]. Our finding might reflect a well-integrated foreign population. This may be due, on the one hand, to the fact that in the last twenty years of immigration foreign citizens learned how to utilize all the services offered by the Italian National Health System and when to access to primary and specialized care rather than to the emergency departments. On the other hand, well-integrated immigrants could be the result of a proper management by medical staff, trained to deal with migrants' specific health problems and able to overcome linguistic and logistic barriers. Immigrants' health concerns are strongly correlated with their country of origin, so that a broad spectrum of variables should be carefully taken into account, when considering both the migration phenomenon and the individual foreign citizen (i.e. nationality, reason of migration such as poverty, political and ethnic persecution or civil war, health standards in the country of origin, social and hygienic conditions in Italy).

Remarkably infectious diseases, initially thought to be a potential prominent problem in these immigrant populations, actually seem to be quite infrequent, when compared with overall morbidity. Immigrant populations are often considered as a source of many known and unknown infectious diseases, such as Ebola, SARS, TBC, malariae, etc. occasionally resulting in unjustified prejudice [18]. As a whole, in our survey infectious diseases accounted for 3.2% of foreign children visits and for 2.5% Italian children visits. Therefore immigrants should not be considered as infectious diseases carriers. Both among immigrant and Italian patients the most frequent presenting problems for visits to emergency departments were respiratory diseases, injury, and digestive symptoms as observed in other cross-sectional study [13], demonstrating that foreign patients and Italian ones suffer by the same pathologies.

## Conclusion

In conclusion, it is not possible to identify a specific "migrant type", remaining the same from a health care, social, economic, and anthropologic point of view. Several different features are typical of each immigrant, and they are generally consistent with the area of origin. Other differences emerge after the immigration process, and they are strictly related to life-style in Italy (i.e. hygienic conditions, nutritional status, etc). Therefore, specific knowledge is needed to face immigrants' different health problems. Under the perspective to better understand foreign patients health needs many instruments, such as translators and multilingual fliers concerning health problems, have to be used in emergency departments.

## Competing interests

The authors declare that they have no competing interests.

## Authors' contributions

ECG participated in the design of the study, performed the statistical analysis and draft the manuscript. CG participated in the design of the study, carried out the data and performed the statistical analysis. AM performed the statistical analysis and draft the manuscript. PDP participated in the design of the study and collection of data. GB conceived of the study and participated in its coordination and helped to draft the manuscript.

All authors read and approved the final version of the manuscript.
